# Pharmacokinetics and safety of the two oral cefaclor formulations in healthy chinese subjects in the fasting and postprandial states

**DOI:** 10.3389/fphar.2022.1012294

**Published:** 2022-10-05

**Authors:** Xinyao Qu, Qiaohuan Deng, Ying Li, Peng Li, Guangwen Liu, Yanli Wang, Zhengzhi Liu, Shuang Yu, Yang Cheng, Yannan Zhou, Jiahui Chen, Qing Ren, Zishu Yu, Zhengjie Su, Yicheng Zhao, Haimiao Yang

**Affiliations:** ^1^ Phase I Clinical Trial Laboratory, Affiliated Hospital of Changchun University of Chinese Medicine, Jilin, China; ^2^ Disha Pharmaceutical Group Co., Ltd., Shanghai, China; ^3^ Shanghai Xihua Scientific Co., Ltd., Shanghai, China; ^4^ Puheng Technology Co., Ltd., Suzhou, China

**Keywords:** antibiotic, equivalence, cefaclor, cephalosporin, pharmacokinetic

## Abstract

We conducted a phase I bioequivalence trial in healthy Chinese subjects in the fasting and postprandial states. The goal of this trial was to compare the pharmacokinetics and safety of the test preparation Cefaclor granule (Disha Pharmaceutical Group Co., Ltd.) and the reference preparation Cefaclor suspension (Ceclor^®^, Eli Lilly and Company). In this trial, 24 subjects were selected in the fasting and postprandial states, respectively. Enrolled subjects randomly accepted a single dose of 0.125 g Cefaclor granule or Cefaclor suspension. The washout period was set as 2 days. Blood samples were collected within 8 h after administration in the fasting state and within 10 h after administration in the postprandial state. Plasma concentrations were determined by Liquid chromatography-tandem mass spectrometry (LC-MS/MS). Pharmacokinetic parameters (AUC, C_max_) were used to evaluate bioequivalence of the two drugs. In the fasting trial, the geometric mean ratios (90% confidence intervals CIs) for C_max_, AUC_0-t_, and AUC_0-∞_ were 93.01% (85.96%–100.63%), 97.92% (96.49%–99.38%) and 97.95% (96.52%–99.41%), respectively. The GMR (90% CIs) for C_max_, AUC_0-t_, and AUC_0-∞_ in postprandial state were 89.27% (81.97%–97.22%), 97.31% (95.98%–98.65%) and 97.31% (95.93%–98.71%), respectively. The 90% CIs of AUC and C_max_ in the fasting and postprandial states were within the 80–125% bioequivalence range. Therefore, Cefaclor granule and Cefaclor suspension were bioequivalent and displayed similar safety profiles. Furthermore, food intake affected the pharmacokinetic parameters of both drugs.

## Introduction

Cefaclor, a ß-lactam antibiotic, is a second-generation cephalosporin antibiotic (Arsalan et al., 2017; Jeong et al., 2021). Cefaclor is considered as a broad-spectrum antibiotic that is effective against both Gram-positive and negative microorganisms such as *Haemophilus* influenzae and *Klebsiella*. It has a strong inhibitory effect on certain anaerobic microorganisms including Propionibacterium acnes (Wilson, 1993; Rai et al., 2010). Cefaclor is widely used in clinical to treat a variety of bacterial infections, involving otitis media, lower respiratory tract infection, upper respiratory tract infection, urinary tract infection, skin infection and sinusitis (Meyers, 2000; Sader et al., 2007; Chen et al., 2012). The anti-bactericidal activity of cefaclor is superior to other first and second generation cephalosporins (Arsalan et al., 2017).

Human pharmacokinetic (PK) studies have shown that cefaclor is well absorbed from the intestinal tract after oral administration (Sourgens et al., 1997; Chen et al., 2012). A single dose of 250 mg cefaclor reaches maximum plasma concentrations within 30–60 min, with a plasma half-life of 0.6–0.9 h (Meyers et al., 1978). At the same time, the effect of food on the absorption of oral cefaclor had also been reported, which might affect the absorption rate of oral cefaclor (Welling and Tse, 1982; Barbhaiya et al., 1990a; Barbhaiya et al., 1990b; Barbhaiya et al., 1990c; Lode et al., 1992).

Here, we conducted a phase I clinical trial to compare the pharmacokinetics and safety of Cefaclor granule and Cefaclor suspension in healthy Chinese subjects in the fasting and postprandial states. Meanwhile, we also focused on the effect of food intake for Cefaclor granule and Cefaclor suspension absorption.

## Materials and methods

### Study subjects

The volunteers were healthy Chinese men and women between the age of 18 and 55 years. The following screening protocols were performed in all volunteers: medical history, physical examination, blood cell count, general biochemistry (including liver function, urine routine), urine pregnancy test for women, HIV, hepatitis B and C virus serological tests, electrocardiography and imaging examinations. Excluded volunteers with one of the exclusion criteria, and the volunteers who met all of the inclusion criteria were enrolled the trial. The detailed inclusion and exclusion criteria were listed in Supplementary materials. 1.

### Study design

This clinical trial was performed at the Phase I Clinical Trial Laboratory, Affiliated Hospital of Changchun University of Chinese Medicine (China). It was conducted according to the requirements of Good Clinical Practice (GCP), the Declaration of Helsinki and the relevant domestic laws and regulations and was approved by the Ethics Committee of the Affiliated Hospital of Changchun University of Chinese Medicine (Number: CCZYFYLL2019 review-006). The study was registered at Drug Clinical Trial Registration and Information Disclosure Platform (Registration No. CTR20190515). All subjects had written informed consents before the initiation of the investigation.

In the fasting state, subjects were randomly divided into two groups (1:1) to receive a single dose of 0.125 g Cefaclor granule (specification: 0.125 g, manufacturer: Disha Pharmaceutical Group Co., Ltd., Shandong, China, batch number: 190102) or Cefaclor suspension (Ceclor^®^, specification: 0.125 g, manufacturer: Eli Lilly and Company, Indianapolis, United States, batch number: C873035). After 2 days washout period, subjects orally administrated another formulation cefaclor with a single dose of 0.125 g. In each administration period, blood samples were collected to assay PK parameters at the following times: within 60 min (pre-dose), 5, 10, 20, 30, 45 min, 1, 1.25, 1.5, 1.75, 2, 3, 4, 6, 8 h after drug administration. The same administration protocol was used in the postprandial state, but blood samples for PK analysis were collected at the following times: within 60 min (pre-dose), 5, 10, 20, 30, 45 min, 1 h, 1 h 20 min, 1 h 40 min, 2 h, 2 h 20 min, 2 h 40 min, 3, 3.5, 4, 6, 8, 10 h after drug administration. 4 mL blood was taken in tubes containing heparin sodium anticoagulant for separation. Blood samples were centrifuged for 10 min at 2000 ± 10 g (4°C) to take plasma using a Beckman Allegra X-15R (Beckman Coulter, Inc., California, UNITED STATES), 800uL plasma was added into the detected tube. All plasma was stored at −80°C within 24 h for preservation until PK analyzed.

### Analytical assays

Plasma samples were analyzed by Liquid Chromatography-Mass Spectrometry (LC-MS/MS) methods using Shimadzu LC-30AD (Kyoto, Japan). The linear quantification ranged from10 ng/mL to 8,000 ng/mL, and the lower limit of quantification was 10 ng/mL. The compounds were separated on a Acquity Uplc Hss T3 column (1.8 µm, 2.1 × 50 mm) at 40°C. Chromatographic and mass spectrometric conditions were as follows. Chromatographic separation was carried out by gradient elution with mobile phase A (0.1% formic acid in water, formic acid purchased from Merck) and mobile phase B (0.1% formic acid in acetonitrile, acetonitrile purchased from Fisher). The flow rate was 0.6 mL/min and the column pressure was 6,000 psi. The injection volume of the sample was 10 µL. Plasm samples were quantified on an API 4000 mass spectrometer using ESI in positive ion mode and multiple reaction monitoring (MRM) using characteristic parent. The mass spectrometry conditions: ion source temperature was 550°C; curtain gas (CUR) was 10 psi; ion source gas 1 (GAS1) was 40 psi; ion source gas 2 (GAS2) was 40 psi; collision gas (CAD) was 6 uint; ion spray voltage (IS) was 5500 V; entrance potential (EP) was10 V; and collision cell exit potential (CXP1) was15 V. The dwelling time was set at 100 msec. The MRM transitions of cefaclor and Cefaclor-d5 (purchased from TLC Pharmaceutical Standards, batch number: 1900-026A3) were m/z 368.1 to 174.1 and m/z 373.1 to 179.1.

Standard solutions and the pre-sample were prepared for the determination of plasma cefaclor concentrations. The standard working solution was used to spike blank plasma samples for the calibration standards at concentrations of 10, 20, 50, 200, 1000,4800, 7200, and 8,000 ng/mL or 10, 30, 300, 4000, 6,400, 32000 ng/mL quality control samples.

### Pharmacokinetic analysis

This clinical trial was a bioequivalence study with pharmacokinetic parameters as the endpoint. The primary endpoint PK parameters were the peak concentration (C_max_), area under the curve (AUC) from time zero to the last measurable concentration (AUC_0-t_) and AUC from time zero to observed infinity (AUC_0-∞_). Further endpoint parameters were the time of the maximum plasma concentration (T_max_), terminal half-life of the analyte in plasma (t_1/2_), terminal rate constant (λ_z_).

### Safety analysis

For safety evaluation, vital signs and adverse events were recorded through a questionnaire. At the end of the trial, blood cell count and general biochemical were given for all participants. The severity of AEs was graded according to the National Cancer Institute Common Terminology Criteria for Adverse Events (NCI CTCAE 5.0). All AEs were recorded throughout the trial and followed until the AEs were eliminated or stabilized.

### Sample size and statistical methods

Based on Guidance for industry bioavailability and bioequivalence studies of drug administration in an account of previous clinical trials, 18–24 subjects can meet the sample size requirements for most drugs, but for some drugs with high variability, the number of subjects should be increased appropriately (European Medicines Agency, 2001; CDER, 2002; CDER, 2021). Previous reports suggest that Cefaclor suspension is not a high variability drug (Meyers et al., 1978; Chen et al., 2012). Considering the number of subjects who could not completed the trial, 24 subjects were enrolled in the fasting and postprandial states, respectively.

### Statistical analysis

Plasma concentration data were analyzed using Phoenix WinNonlin software 7.0 (Phoenix WinNonlin, Certara United States, Inc., Princeton, NJ, United States) and PK parameters were calculated including C_max_, AUC_0-t_, AUC_0-∞_, T_max_, λ_Z_, t_1/2_. The plasma drug concentration curve was log-transformed by Prism software 8.0 (GraphPad Software, San Diego, CA, United States). All data statistical analysis were performed using SAS software 9.4 (SAS Institute Inc., Cary, NC, United States). Major pharmacokinetic parameters C_max_, AUC_0-t_ and AUC_0-∞_ were transformed natural logarithm and tested for significance by analysis of variance (ANOVA). Statistical analysis using two-side test and 90% confidence interval was used to evaluate the bioequivalence of the test and reference drug. When the 90% CI of C_max_, AUC_0-t_ and AUC_0-∞_ GMR for two drugs were in the bioequivalent interval of 80.00–125.00%, the two drugs were considered to be bioequivalent.

## Results

### Subject demographics

**FIGURE 1:**
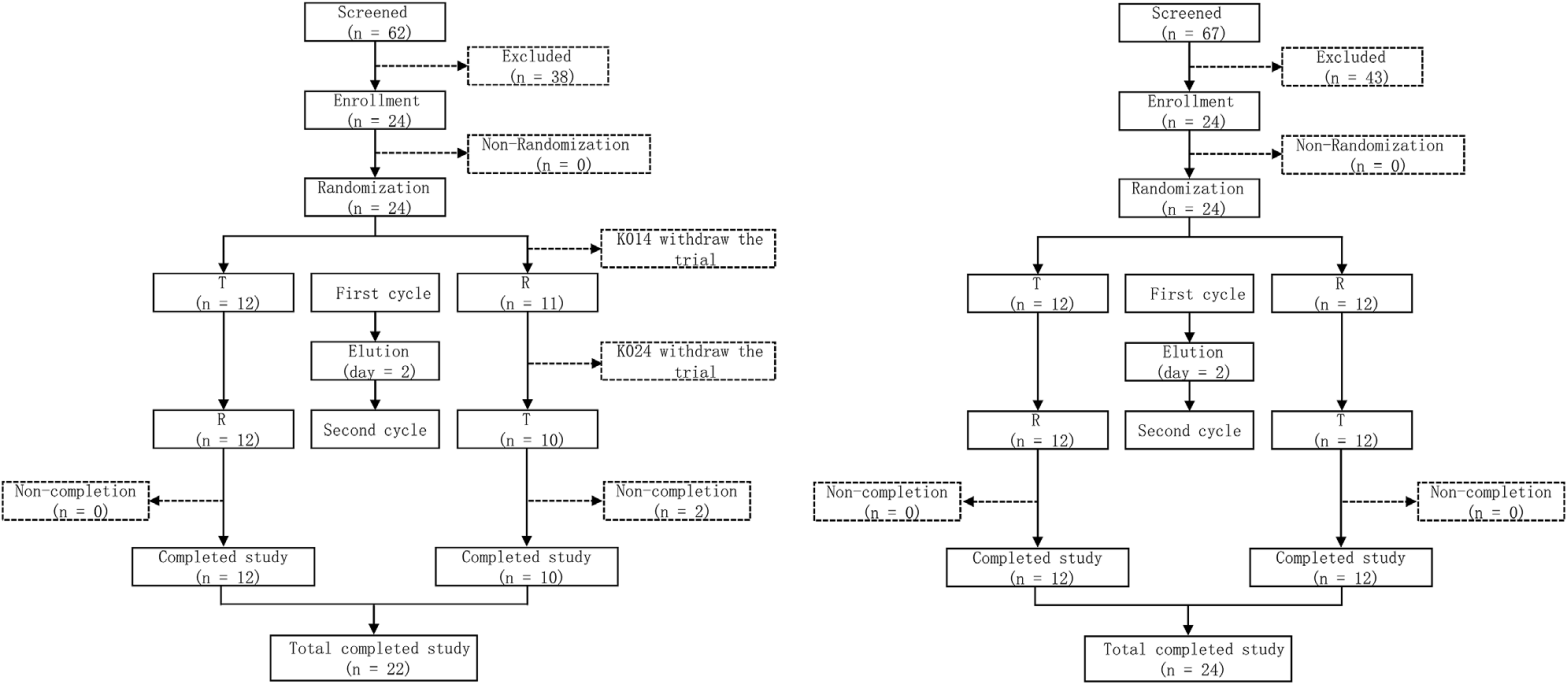
Study flow diagram under the fasting state and the postprandial state. T, test drug was Cefaclor granule; R, reference drug was Cefaclor suspension; n, number of subjects.

In the trial screening for fasting and postprandial states, 62 and 64 volunteers participated, respectively. Ultimately, the trial regarding the fasting state and the postprandial state enrolled 24 subjects, respectively. Two subjects belonging to fasting state have withdrawn from the trial. NO. K014 discontinued the trial due to vomiting within the double times of medium T_max_. The blood samples were collected prior to the first dosing period. NO. K024 withdrew from the trial due to adverse events (sweating, slow pulse). The blood samples were collected within 2 h after Cefaclor suspension administration. Besides, the two subjects were still enrolled in the full analysis set (FAS) and safety analysis set (SS), the No. K014 was not enrolled in pharmacokinetics analysis set (PKPS) and bioequivalence analysis set (BES), the No. K024 was enrolled in PKPS but not in BES. In the postprandial state, 24 subjects were all included in the safety analysis set, and no adverse events led subject withdrawn from the trial. The detailed process for this trial is shown in Figure 1. The basic information about the subjects is shown in Table 1. There were no significant differences in demographic characteristics between the two sequences. Subjects who participated in the trial were all up to the inclusion criteria.

**TABLE 1:** Demographic and baseline characteristics of the study participants.

	Fasting sequence (N = 24)	Postprandial sequence (N = 24)
Age (years)		
Mean ± SD	38.96 ± 10.89	36.88 ± 9.19
Median (Q1, Q3)	42.5 (28.5, 48)	37.0 (28.5, 45)
Min–Max	19.0–53.0	23.0–50.0
Sex n (%)		
Male	13 (54.17%)	13 (54.17%)
Female	11 (45.83%)	11 (45.83%)
Ethnicity n (%)		
Ethnic Han	23 (95.83%)	24 (100%)
Others	1 (4.17%)	0 (0)
Height (cm)		
Mean ± SD	164.85 ± 8.41	167.23 ± 8.29
Median (Q1, Q3)	166.25 (159, 171)	165.5 (161.25, 174.75)
Min–Max	148.0–178.5	151.5–182
Weight (kg)		
Mean ± SD	64.72 ± 9.99	67.68 ± 9.69
Median (Q1, Q3)	61.6 (56.85, 72.45)	68.25 (61.4, 74.7)
Min–Max	48.7–81.3	49.3–84.2
BMI (kg/m^2^)		
Mean ± SD	23.73 ± 2.45	24.12 ± 2.2
Median (Q1, Q3)	23.55 (22.2, 25.85)	23.95 (22.8, 26.15)
Min–Max	18.4–27.8	18.5–27.8

N, number of subjects; SD, standard deviation; Q1, 1st quartile; Median, 2nd quartile; Q3, 3rd quartile; BMI, body mass index [defined as weight/(height in meters)^2^].

### Pharmacokinetic results

**FIGURE 2:**
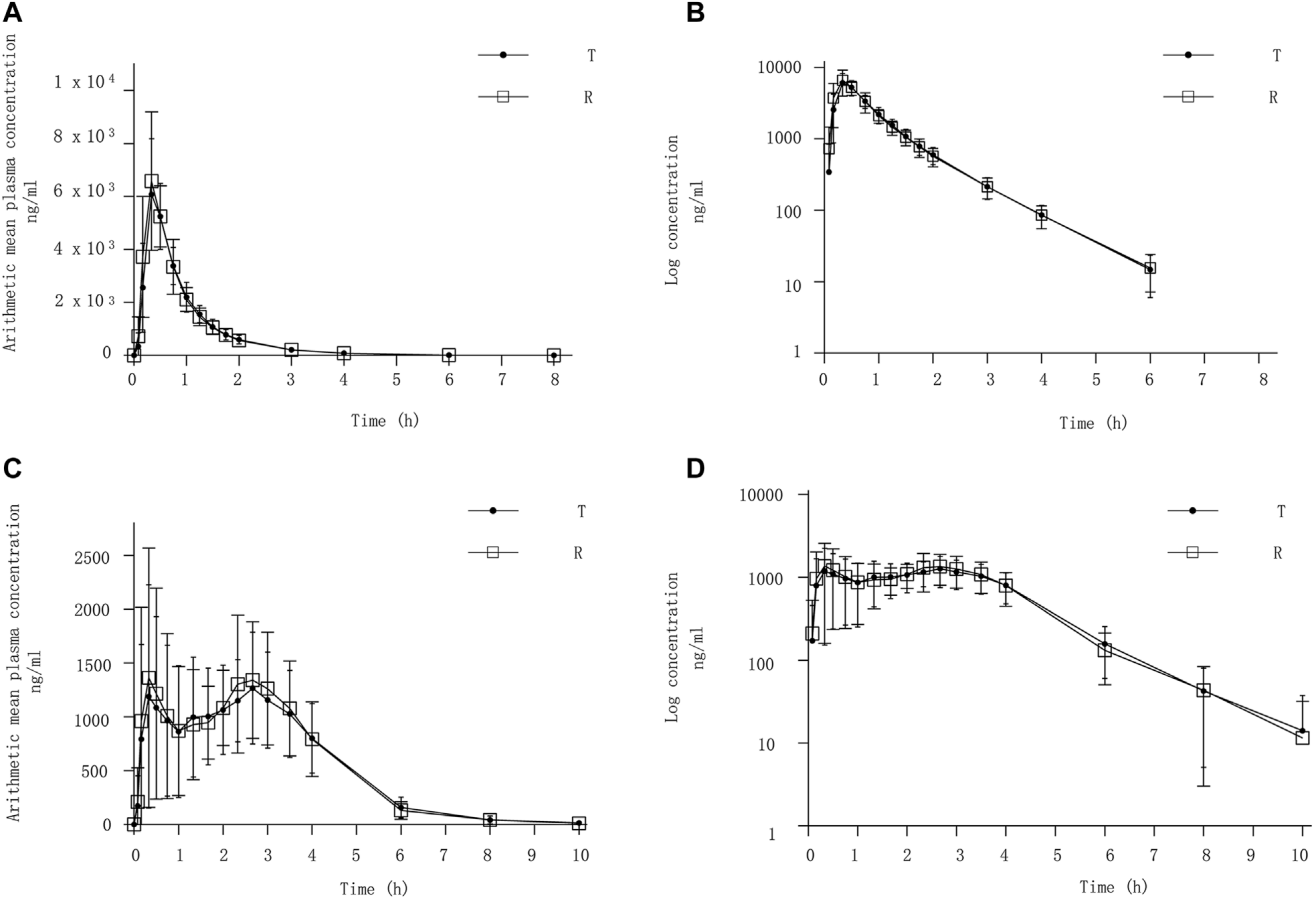
PK analysis of Cefaclor granule and Cefaclor suspension. T, test drug was Cefaclor granule; R, reference drug was Cefaclor suspension; Mean blood concentration (±SD) time curve after oral administrated Cefaclor granule and Cefaclor suspension during fasting: arithmetic mean **(A)** and log transformation **(B)**. Mean blood concentration (±SD) time curve after oral Cefaclor granule and Cefaclor suspension in postprandial state: arithmetic mean **(C)** and log transformation **(D)**.

After completing 2 periods of administration, the mean ± SD plasma concentration-time curve for Cefaclor granule and Cefaclor suspension in the fasting state is shown in Figure 2A. The curve after logarithmic transformation is shown in Figure 2B. The mean ± SD (CV%) of the C_max_ values for Cefaclor granule and Cefaclor suspension were 6,432.73 ± 1645.99 ng/mL (25.59%) and 7006.52 ± 2032.34 ng/mL (29.01%), respectively; the AUC_0-t_ values were 5421.56 ± 1013.57 h*ng/mL (18.70%) and 5548.92 ± 1116.70 h*ng/mL (20.12%), respectively; and the AUC_0-∞_ values were 5446.43 ± 1013.11 h*ng/mL (18.60%) and 5573.02 ± 1118.52 h*ng/mL (20.07%), respectively. The median T_max_ of the two drugs were 0.3333 h and 0.3331 h, respectively. The mean ± SD of the t_1/2_ values for Cefaclor granule and Cefaclor suspension were 0.78 ± 0.10 h and 0.79 ± 0.08 h. The mean ± SD of the λ_z_ values for Cefaclor granule and Cefaclor suspension were 0.91 ± 0.12 and 0.89 ± 0.09. Other detailed PK parameters are listed in Table 2. The results of ANOVA for primary PK parameters indicated that no significant difference in C_max_ for drug formulations (Cefaclor granule and Cefaclor suspension), administration periods and sequences (*p* > 0.05). Although AUC_0-t_ and AUC_0-∞_ were not statistically different during the administration periods (*p* > 0.05), there were statistical differences between drug formulations and administration sequences (*p* < 0.05) (Supplementary Table S1).

**TABLE 2:** Summary of pharmacokinetic parameters after oral two drugs in fasting and postprandial sequences.

PK parameters	Fasting sequence		Postprandial sequence	
	Cefaclor granule ^b^	Cefaclor suspension ^b^	Cefaclor granule	Cefaclor suspension
C_max_ (ng/mL)				
N (Missing)	22 (0)	23 (0)	24 (0)	24 (0)
Mean ± SD	6,432.73 ± 1645.99	7006.52 ± 2032.34	1982.50 ± 601.31	2276.25 ± 831.71
Median (Q1, Q3)	6,470.0 (5020.0–7560.0)	7280.0 (4850.0–7900.0)	1880.0 (1665.0–2155.0)	1840.00 (1770.0–2825.0)
Min-Max	3790.0–9430.0	4280.0–11500.0	1140.0–4190.0	1240.0–3980.0
T_max_ (h)				
N (Missing)	22 (0)	23 (0)	24 (0)	24 (0)
Mean ± SD	0.42 ± 0.13	0.38 ± 0.08	1.73 ± 1.21	1.69 ± 1.23
Median (Q1, Q3)	0.3333 (0.3322–0.4983)	0.3331 (0.3322–0.4969)	1.9997 (0.3329–2.6644)	2.168 (0.3333–2.6648)
Min-Max	0.3306–0.7483	0.3297–0.4989	0.1647–3.9983	0.1664–3.4997
AUC_0-t_ (h*ng/mL)				
N (Missing)	22 (0)	22 (1) ^a^	24 (0)	24 (0)
Mean ± SD	5421.56 ± 1013.57	5548.92 ± 1116.70	5253.26 ± 669.95	5396.25 ± 661.68
Median (Q1, Q3)	5275.13 (4831.20–6,054.11)	5379.47 (4771.96–6,298.42)	5195.32 (4663.40–5613.74)	5379.45 (4819.23–5935.88)
Min-Max	3500.56–7810.22	3760.36–8,071.93	4124.47–6,468.16	4145.30–6,724.68
AUC_0-∞_ (h*ng/mL)				
N (Missing)	22 (0)	22 (1) ^a^	24 (0)	24 (0)
Mean ± SD	5446.43 ± 1013.11	5573.02 ± 1118.52	5299.69 ± 691.82	5442.40 ± 671.45
Median (Q1, Q3)	5289.73 (4843.23–6,079.71)	5394.24 (4787.16–6,332.56)	5222.10 (4710.78–5693.83)	5409.44 (4835.19–5965.71)
Min-Max	3542.04–7846.02	3811.80–8,104.21	4147.13–6,610.61	4177.31–6,927.89
λ_z_ (1/h)				
N (Missing)	22 (0)	22 (1) ^a^	24 (0)	24 (0)
Mean ± SD	0.91 ± 0.12	0.89 ± 0.09	0.78 ± 0.23	0.76 ± 0.24
Median (Q1, Q3)	0.87 (0.84–1.04)	0.88 (0.80–0.96)	0.76 (0.59–0.97)	0.75 (0.62–0.95)
Min-Max	0.73–1.14	0.76–1.07	0.40–1.18	0.21–1.17
t_1/2_ (h)				
N (Missing)	22 (0)	22 (1) ^a^	24 (0)	24 (0)
Mean ± SD	0.78 ± 0.10	0.79 ± 0.08	0.98 ± 0.34	1.05 ± 0.56
Median (Q1, Q3)	0.79 (0.67–0.83)	0.78 (0.72–0.87)	0.91 (0.72–1.19)	0.92 (0.73–1.12)
Min-Max	0.61–0.95	0.65–0.91	0.59–1.71	0.59–3.27

N, number of subjects; C_max_, The maximum observed drug concentration in the plasma; T_max_, the time from administration to the maximum observed concentration of the analyte in the plasma; AUC _0-t_, the AUC of the analyte in the plasma over the time interval from time zero to the last measurable concentration; AUC_0-∞_, the area under the curve from 0 to infinity; λ_z:_ terminal rate constant in the plasma; T_1/2_, the terminal half-life of the analyte in the plasma.

a Subject with randomized number K024 discontinued the trial due to AEs 2 hours after oral administration Cefaclor suspension. Thus, PKPS only recorded T_max_ and C_max_, while AUC_0-t_, AUC_0-∞_, t_1/2_ and λ_Z_ were absent.

b Subject with randomized number K014 discontinued the trial due to vomiting within the double medium of T_max_ of Cefaclor suspension. Only blood samples were collected prior to the first dosing period, so subject participated in SS but not PKPS and BES. Subject with random number K024 discontinued the trial due to AEs. Blood samples were collected within 2 hours after oral administration Cefaclor suspension, therefore, the subject enrolled in SS and PKPS but not BES.

In the postprandial state, the mean ± SD plasma concentration-time curve for Cefaclor granule and Cefaclor suspension is shown in Figure 2C. The curve after logarithmic transformation is shown in Figure 2D. The mean ± SD (CV%) of the C_max_ values for Cefaclor granule and Cefaclor suspension were 1982.50 ± 601.31 ng/mL (30.33%) and 2276.25 ± 831.71 ng/mL (36.54%), respectively; the AUC_0-t_ values were 5253.26 ± 669.95 h*ng/mL (12.75%) and 5396.25 ± 661.68 h*ng/mL (12.26%), respectively; and the AUC_0-∞_ values were 5299.69 ± 691.82 h*ng/mL (13.05%) and 5442.40 ± 671.45 h*ng/mL (12.34%), respectively. The median T_max_ of the two drugs were 1.9997 h and 2.168 h, respectively. The mean ± SD of the t_1/2_ values for Cefaclor granule and Cefaclor suspension were 0.98 ± 0.34 h and 1.05 ± 0.56 h. The mean ± SD of the λ_z_ values for Cefaclor granule and Cefaclor suspension were 0.78 ± 0.23 and 0.76 ± 0.24. Other detailed PK parameters are listed in Table 2. The results of ANOVA for primary PK parameters showed that there were no significant differences in C_max_, AUC_0-t_ and AUC_0-∞_ for administration periods and sequences (*p* > 0.05). However, both C_max_, AUC_0-t_ and AUC_0-∞_ had statistical differences with drug formulations (*p* < 0.05) (Supplementary Table S1).

The above PK parameters suggested that food intake might affect C_max_ and T_max_ but not AUC. Meanwhile, there were no differences in the PK parameters values between Cefaclor granule and Cefaclor suspension (*p* > 0.05).

### Bioequivalence results

**FIGURE 3:**
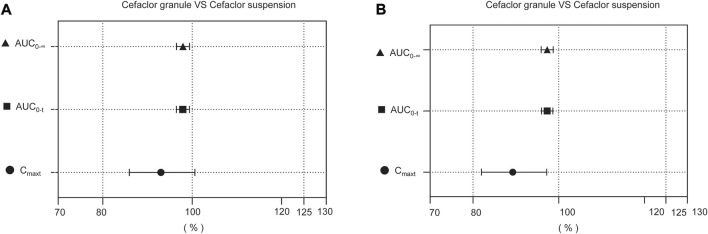
Bioequivalence analysis of Cefaclor granule and Cefaclor suspension. The bioequivalence analysis of Cefaclor granule and Cefaclor suspension during fasting **(A)** shows the ratio range of the main PK parameters of Cefaclor granule and Cefaclor suspension with 90% CI. The bioequivalence analysis of Cefaclor granule and Cefaclor suspension in postprandial state **(B)** shows the ratio range of the main PK parameters with 90% CI (bioequivalence was declared if the 90% CIs were within the prespecified acceptable ranges of 80–125%). AUC0-t: AUC of the analyte in the plasma over the time interval from time zero to the last measurable concentration; AUC0-∞: AUC of the analyte in the plasma over the time interval from time zero to infinity; Cmax: maximum observed drug concentration in the plasma.

The comparisons of the geometric mean ratios (GMRs) for the main pharmacokinetic parameters between Cefaclor granule and Cefaclor suspension in the fasting state are listed in Table 3. In the fasting state, the GMRs values (power) of C_max_, AUC_0-t_, and AUC_0-∞_ for Cefaclor granule and Cefaclor suspension were 93.01% (93.78%), 97.92% (>99.99%) and 97.95% (>99.99%), respectively. The 90% confidence intervals (CIs) of GMRs for C_max_, AUC_0-t_, and AUC_0-∞_ were 85.96%–100.63%, 96.49%–99.38% and 96.52%–99.41%, respectively. The primary PK parameters were within the bioequivalence range of 80.00–125.00%. The above results indicated that Cefaclor granule was bioequivalent to Cefaclor suspension in the fasting state (Figure 3A).

**TABLE 3:** Results of the bioequivalence determination of Cefaclor granule and Cefaclor suspension in fasting sequence.

PK parameter		Geometric mean	GMR (%)	90% CI (%)	%CV	Power	Result
C_max_ (ng/mL)	T (N = 22)	6272.71	93.01	85.96–100.63	15.18	93.78	Bioequivalent
	R (N = 22)	6744.49					
AUC_0-t_ (h_*_ng/mL)	T (N = 22)	5383.22	97.92	96.49–99.38	2.83	>99.99	
	R (N = 22)	5497.41					
AUC_0-∞_ (h_*_ng/mL)	T (N = 22)	5409.02	97.95	96.52–99.41	2.82	>99.99	
	R (N = 22)	5522.09					

T, test drug was Cefaclor granule; R, reference drug was Cefaclor suspension; PK, Pharmacokinetic; N, number of subjects; GMR, geometric mean ratios; CI, confidence Interval; CV%, within-subject coefficient of variation; AUC _0-t_, the AUC of the analyte in the plasma over the time interval from time zero to the last measurable concentration; AUC_0-∞_, the area under the curve from 0 to infinity; C_max_, the maximum observed drug concentration in the plasma.

The GMRs comparison for the main pharmacokinetic parameters between Cefaclor granule and Cefaclor suspension in the postprandial state are listed in Table 4. The GMRs values (power) of C_max_, AUC_0-t_, and AUC_0-∞_ for Cefaclor granule and Cefaclor suspension were 89.27% (68.92%), 97.31% (>99.99%) and 97.31% (>99.99%), respectively. The 90% CI of GMRs for C_max_, AUC_0-t_, and AUC_0-∞_ were 81.97%–97.22%, 95.98%–98.65% and 95.93%–98.71%, respectively. The primary PK parameters were within the bioequivalence range of 80.00–125.00%. These results showed that Cefaclor granule was bioequivalent to Cefaclor suspension in the postprandial state (Figure 3B).

**TABLE 4:** Results of the bioequivalence determination of Cefaclor granule and Cefaclor suspension in postprandial sequence.

PK parameter		Geometric mean	GMR (%)	90% CI (%)	%CV	Power	Result
C_max_ (ng/mL)	T (N = 24)	1913.19	89.27	81.97–97.22	17.34	68.92	Bioequivalent
	R (N = 24)	2143.11					
AUC_0-t_ (h_*_ng/mL)	T (N = 24)	5213.08	97.31	95.98–98.65	2.77	>99.99	
	R (N = 24)	5357.38					
AUC_0-∞_ (h_*_ng/mL)	T (N = 24)	5257.47	97.31	95.93–98.71	2.88	>99.99	
	R (N = 24)	5402.92					

T, test drug was Cefaclor granule; R, reference drug was Cefaclor suspension; PK, Pharmacokinetic; N, number of subjects; GMR, geometric mean ratios; CI, confidence Interval; CV%, within-subject coefficient of variation; AUC _0-t_, the AUC of the analyte in the plasma over the time interval from time zero to the last measurable concentration; AUC_0-∞_, the area under the curve from 0 to infinity; C_max_, the maximum observed drug concentration in the plasma.

### Safety results

In general, both two drugs exhibited well safety in healthy Chinese subjects after meals or under an empty stomach status. In the fasting states, there were 13 adverse events (AEs) in 11 subjects caused by Cefaclor granule and Cefaclor suspension, such as anemias, vomited, sweating, slow pulse, dizziness, infection urinary tract and positive urinary occult blood. Five subjects had 5 AE cases regarding Cefaclor granule, 6 subjects had 8 AE cases in Cefaclor suspension group. The detailed AEs are shown in Table 5, there were no AEs of grade 3 or above. In the postprandial state, three subjects experienced three grade I drug-related adverse events after Cefaclor granule and Cefaclor suspension administration, such as anemia, infection urinary tract and positive urinary occult blood. There were 2 AE cases in 2 subjects caused by Cefaclor granule, and one subject had one AE in Cefaclor suspension group, the detailed AEs are shown in Table 6.

**TABLE 5:** Summary of AEs for Cefaclor granule and Cefaclor suspension in the fasting trial.

AE category	Cefaclor granule ^a^		Cefaclor suspension ^a^	
	N	n	N	n
Total adverse events	5	5	6	8
AEs related to the study drugs	5	5	6	8
Anemia	1	1	0	0
Vomited	0	0	1	1
Sweating	0	0	1	1
Slow pulse	0	0	1	1
Dizziness	0	0	1	1
Infection urinary tract	2	2	0	0
Urinary occult blood positive	2	2	4	4
AEs of grade 1	5	5	6)	6
AEs of grade 2	0	0	1	2
AEs of grade 3 and above	0	0	0	0
AEs leading to discontinuation of study drug	0	0	2	4

a Subject with random number K014 discontinued the trial due to AEs. Blood samples were collected from subjects prior to oral medication, so subject participated in SS but not PKPS and BES. Subject with random number K024 discontinued the trial due to AEs. Blood samples were collected within 2 hours after oral administration Cefaclor suspension, therefore, the subject enrolled in SS and PKPS but not BES. The number of cefaclor was 22, the number of Cefaclor suspension was 24.

N, number of the subjects with adverse events; n, the number of adverse events; AEs, adverse events.

**TABLE 6:** Summary of AEs for Cefaclor granule and Cefaclor suspension in postprandial trial.

AE category	Cefaclor granule		Cefaclor suspension	
	N	n	N	n
Total adverse events	2	2	1	1
AEs related to the study drugs	2	2	1	1
Anemia	1	1	0	0
Infection urinary tract	0	0	0	0
Urinary occult blood positive	1	1	1	1
AEs of grade 1	2	2	1	1
AEs of grade 2	0	0	0	0
AEs of grade 3 and above	0	0	0	0
AEs leading to discontinuation of study drug	0	0	0	0

N, number of the subjects with adverse events; n, the number of adverse events; AEs, adverse events.

## Discussion

Cefaclor is a highly absorbable oral cephalosporin antibiotic widely used in outpatient treatment for community-acquired pneumonia and other mild to moderate infections (Sader et al., 2007). This trial was designed to compare the bioequivalence and safety of Cefaclor granule and Cefaclor suspension under fasting and postprandial states. Meanwhile, the effect of food intake on Cefaclor granule and Cefaclor suspension PK parameters was also investigated. The fasting and postprandial study sequences were two separate dosing sequences. The trial used a 2 × 2 cross-over study design which was up to the requirements of a bioequivalence trial. Previous studies indicated that age and gender had no significant effects on the PK parameters of Cefaclor granule and Cefaclor suspension (Satterwhite et al., 1992; Nix et al., 1997). Therefore, this study recruited male and female subjects between the ages from 18 to 55 years. In the postprandial state, the metabolic half-life of 250 mg oral cefaclor was 1–1.5 h, to avoid the influence of the previous administration of induced residue, the washout period of this trial was set as 2 days (Karim et al., 2003; FDA, 2021). The washout period was 7 times more than the drug metabolic half-life, which was enough to ensure that the drug concentration before the next administration was lower than the lower limit of the bioassay quantitation. In the fasting trial, the plasma concentrations of several samples exceeded the linear quantitative range (10.0 ng/mL to 8,000 ng/mL). Thus, we diluted the samples 5-fold for detection. 32000 ng/mL is the diluted QC sample concentrate and higher than the upper limit of quantification (8,000 ng/mL). The diluted QC was detected after a 5-fold dilution (6,400 ng/mL) which is within the quantitative linear range. The QC results met the 15% acceptance criteria and the residue was also acceptable for common compliance.

The pharmacokinetic parameter values for Cefaclor suspension in this trial were very similar to previously published data (Glynne et al., 1978; Wilson, 1993; Chen et al., 2012). At the same time, available data suggested that dietary substances can alter the absorption rate and efficiency of oral cefaclor (Oguma et al., 1991). There may be some discrepancies between the reported results. The results of the current study were quite different, with C_max_ and AUC of cefaclor showing similar changes after different types of breakfasts (Williams et al., 1996). The result of a report regarding the effects of different foods on absorption of cefaclor shows that food intake did not affect the areas under the concentration-time curves, but reduced the maximum concentration and prolonged the time to maximum concentration of drug in serum (Oguma et al., 1991). In the study of Barbhaiya, R.H., et al., food intake increased the t_max_ of cefaclor compared to fasting condition (Barbhaiya et al., 1990a). In our trial, the Cmax values of Cefaclor granule and Cefaclor suspension were significantly decreased and the Tmax values were significantly increased in the postprandial state. The presence of food did not significantly alter the AUC of cefaclor, although there was a slight decrease in AUC compared to values in the fasting state. In the postprandial group, there were significant differences in C_max_ and AUC between the two formulations. However, the 90% CI for PK parameters ranged from 80% to 125% in this trial under the fasting and postprandial states. Therefore, it can be concluded that Cefaclor granule and Cefaclor suspension were bioequivalent.

Cefaclor has high plasma concentration and a low risk of gastrointestinal side effects due to its rapid and high rate of absorption (compared to other antibiotics, cefaclor reaches peak plasma concentrations within 1 h) (Sides et al., 1988). Subjects were in good general health, with stable vital signs and no SAEs in the trial. AEs occurred in the trial included anemia, vomited, sweating, slow pulse, dizziness, infection urinary tract and positive urinary occult blood. The safety profile of this trial was similar to other bioequivalence trials (Koytchev et al., 2004; Chen et al., 2012). Adverse events associated with cefaclor treatment were rarely reported. Adverse events reported in post-marketing surveillance included allergic reaction, anaphylactoid reaction, angioedema, facial edema, hypotension, Stevens-Johnson syndrome, syncope, paresthesia, vasodilation and vertigo (Meyers, 2000). The influence of other factors cannot be determined.

An interesting phenomenon emerged in Figure 2C, a double peak was found in the plasma concentration-time curve. To determine the cause, we plotted the plasma concentration-time curves for each subject in the postprandial state (Supplementary Figure S1). Plasma concentrations of Cefaclor granule and Cefaclor suspension for each subject in the postprandial state are shown in Supplementary Table S2 and Supplementary Table S3. We research for more literature to better explain this double peak phenomenon for Cefaclor. After a single oral dose of cefaclor, the plasma concentration-time curve of the fasting trial did not show a double-peak phenomenon, but the postprandial trial showed a double-peak phenomenon. Thus, the factor that can cause double peaks is gastric emptying and gastric motility. The residence time of a drug in the gastrointestinal tract affects the rate and extent of drug absorption after oral administration (Nimmo et al., 1975). But residence time is mainly determined by gastric emptying and gastrointestinal motility (Levine, 1970). Changes in gastric emptying and intestinal flow rates after a single dose can lead to changes in absorption rates throughout the course of absorption (Oberle and Amidon, 1987). However, in the previous studies for cefaclor after oral administration we did not find the double peek phenomenon (Barbhaiya et al., 1990a; Oguma et al., 1991; Chen et al., 2012). Further research is needed to determine the cause of this phenomenon.

Based on the results of this trial, the rate and extent of absorption for Cefaclor granule and Cefaclor suspension were comparable in the fasting and postprandial status. There was no significant difference in AUC values ​​between 0.125 g Cefaclor granule and Cefaclor suspension. In contrast, the C_max_ and T_max_ values calculated in the fasting state were approximately 3-fold and 6-fold higher than that in the postprandial state. The PK parameters C_max_ values for the two studies after meal and under an empty stomach status were 6,432.73 ± 1645.99 ng/mL (Cefaclor granule) and 7006.52 ± 2032.34 ng/mL (Cefaclor suspension), as well as 1982.50 ± 601.31 ng/mL (Cefaclor granule) and 2276.25 ± 831.71 ng/mL (Cefaclor suspension), respectively. The results were similar to the data published (Williams and Harding, 1984; Barbhaiya et al., 1990a).

There are several limitations in the current study. First, this study merely demonstrates that the biosimilar is similar to the “originals” in terms of PK parameters. Thus, the therapeutic bioequivalence between the biosimilar and the original needs further trials to verify. The sample size is another limitation of the study, although it is up to the requirements of a bioequivalence trial. Due to sample size limitations, we were unable to conduct a comprehensive assessment for the safety of these two drugs. Lastly, our trial is single-dose administration, and there is no cumulative exposure to the drug in the human body, which will also affect the assessment of drug safety.

## Conclusion

This trial is a single-center, randomized, open, single-dose, two-period crossover phase I clinical trial to compare the bioequivalence and safety of Cefaclor granule (Disha Pharmaceutical Group Co., Ltd.) and Cefaclor suspension (Ceclor^®^, Eli Lilly and Company) in healthy Chinese subjects under the fasting and postprandial states. By evaluating the primary PK parameters, C_max_, AUC_0-t_, and AUC_0-∞_ all met the bioequivalence criteria, supporting the bioequivalence of the two drugs. Cefaclor granule and Cefaclor suspension were safe in healthy Chinese subjects in the fasting and postprandial states.

## Key points

1) The results of this trial indicated that Cefaclor granule and Cefaclor suspension were bioequivalent and displayed similar safety profiles.
2) Food intake reduced the maximum plasma concentration and prolonged the peak time of the two oral cefaclor.

## Data Availability

The original contributions presented in the study are included in the article/Supplementary Material, further inquiries can be directed to the corresponding author.

## References

[B1] Arsalan A. Ahmad I. Ali S. A. (2017). Cefaclor: Clinical, biochemical, analytical and stability aspects. Adv. Med. Biol. 123, 1–52.

[B2] Barbhaiya R. H. Gleason C. R. Shyu W. C. Wilber R. B. Martin R. R. Pittman K. A. (1990). Phase I study of single-dose BMY-28100, a new oral cephalosporin. Antimicrob. Agents Chemother. 34 (2), 202–205. 10.1128/aac.34.2.202 2327766 PMC171556

[B3] Barbhaiya R. H. Shukla U. A. Gleason C. R. Shyu W. C. Pittman K. A. (1990). Comparison of the effects of food on the pharmacokinetics of cefprozil and cefaclor. Antimicrob. Agents Chemother. 34 (6), 1210–1213. 10.1128/aac.34.6.1210 2393283 PMC171786

[B4] Barbhaiya R. H. Shukla U. A. Gleason C. R. Shyu W. C. Wilber R. B. Pittman K. A. (1990). Comparison of cefprozil and cefaclor pharmacokinetics and tissue penetration. Antimicrob. Agents Chemother. 34 (6), 1204–1209. 10.1128/aac.34.6.1204 2393282 PMC171785

[B5] CDER (2002). Guidance for industry bioavailability and bioequivalence studies for orally administered drug products-general considerations. Available from https://docslib.org/doc/1597843/guidance-for-industry-bioavailability-and-bioequivalence-studies-for.

[B6] CDER (2021). Guidance for industry, statisitical approaches to estabalishing bioequivalence,January. Available from: https://www.fda.gov/media/70958/download.

[B7] Chen J. Jiang B. Lou H. Yu L. Ruan Z. (2012). Bioequivalence studies of 2 oral cefaclor capsule formulations in Chinese healthy subjects. Arzneimittelforschung. 62 (3), 134–137. 10.1055/s-0031-1298012 22286978

[B8] European Medicines Agency (2001). Notes for guidance on the investigation of bioavailability and bioequivalence. Available from: https://www.ema.europa.eu/en/documents/scientific-guideline/guideline-investigation-bioequivalence-rev1_en.pdf.

[B9] FDA (2021) Ceclor® FDA approved information. Available at: https://www.accessdata.fda.gov/scripts/cder/daf/index.cfm?event=overview.process&ApplNo=050522.

[B10] Glynne A. Goulbourn R. A. Ryden R. (1978). A human pharmacology study of cefaclor. J. Antimicrob. Chemother. 4 (4), 343–348. 10.1093/jac/4.4.343 690034

[B11] Jeong S. H. Jang J. H. Cho H. Y. Lee Y. B. (2021). Population pharmacokinetic analysis of cefaclor in healthy Korean subjects. Pharmaceutics 13 (5), 754. 10.3390/pharmaceutics13050754 34069627 PMC8160640

[B12] Karim S. Ahmed T. Monif T. SahaN. Sharma P. L. (2003). The effect of four different types of food on the bioavailability of cefaclor. Eur. J. Drug Metab. Pharmacokinet. 28 (3), 185–190. 10.1007/BF03190484 14527091

[B13] Koytchev R. Ozalp Y. Erenmemisoglu A. Tyutyulkova N. Gatchev E. Alpan R. S. (2004). Studies on the bioequivalence of second generation cephalosporins: Cefaclor capsules and suspension. Arzneimittelforschung. 54 (9A), 583–587. 10.1055/s-0031-1297053 15497664

[B14] Levine R. R. (1970). Factors affecting gastrointestinal absorption of drugs. Am. J. Dig. Dis. 15 (2), 171–188. 10.1007/BF02235648 4905589

[B15] Lode H. Muller C. Borner K. Nord C. E. KoePPe P. (1992). Multiple-dose pharmacokinetics of cefprozil and its impact on intestinal flora of volunteers. Antimicrob. Agents Chemother. 36 (1), 144–149. 10.1128/aac.36.1.144 1590680 PMC189242

[B16] Meyers B. R. (2000). Cefaclor revisited. Clin. Ther. 22 (2), 154–166. 10.1016/S0149-2918(00)88477-5 10743978

[B17] Meyers B. R. Hirschman S. Z. Wormser G. GartenberG G. SrulEvitch E. (1978). Pharmacologic studies with cefaclor, a new oral cephalosporin. J. Clin. Pharmacol. 18 (4), 174–179. 10.1002/j.1552-4604.1978.tb01590.x 632363

[B18] Nimmo W. S. Heading R. C. Wilson J. Tothill P. Prescott L. F. (1975). Inhibition of gastric emptying and drug absorption by narcotic analgesics. Br. J. Clin. Pharmacol. 2 (6), 509–513. 10.1111/j.1365-2125.1975.tb00568.x 9953 PMC1402648

[B19] Nix D. E. Symonds W. T. Hyatt J. M. Wilton J. H. Teal M. A. Reidenberg P. (1997). Comparative pharmacokinetics of oral ceftibuten, cefixime, cefaclor, and cefuroxime axetil in healthy volunteers. Pharmacotherapy 17 (1), 121–125. 9017772

[B20] Oberle R. L. Amidon G. L. (1987). The influence of variable gastric emptying and intestinal transit rates on the plasma level curve of cimetidine; an explanation for the double peak phenomenon. J. Pharmacokinet. Biopharm. 15 (5), 529–544. 10.1007/BF01061761 3694496

[B21] Oguma T. Yamada H. SawakiM. NaritaN. (1991). Pharmacokinetic analysis of the effects of different foods on absorption of cefaclor. Antimicrob. Agents Chemother. 35 (9), 1729–1735. 10.1128/aac.35.9.1729 1952839 PMC245259

[B22] Rai A. Prabhune A. Perry C. C. (2010). Antibiotic mediated synthesis of gold nanoparticles with potent antimicrobial activity and their application in antimicrobial coatings. J. Mat. Chem. 20 (32), 6789–6798. 10.1039/c0jm00817f

[B23] Sader H. S. Jacobs M. R. Fritsche T. R. (2007). Review of the spectrum and potency of orally administered cephalosporins and amoxicillin/clavulanate. Diagn. Microbiol. Infect. Dis. 57, 5S–12S. 10.1016/j.diagmicrobio.2006.12.014 17292577

[B24] Satterwhite J. H. Cerimele B. J. Coleman D. L. Hatcher B. L. Kisicki J. DeSante K. A. (1992). Pharmacokinetics of cefaclor AF: Effects of age, antacids and H2-receptor antagonists. Postgrad. Med. J. 68, S3–S9. 1287615

[B25] Sides G. Franson T. R. DeSante K. A. Black H. R. (1988). A comprehensive review of the clinical pharmacology and pharmacokinetics of cefaclor. Clin. Ther. 11, 5–19. 3072086

[B26] Sourgens H. Derendorf H. Schifferer H. (1997). Pharmacokinetic profile of cefaclor. Int. J. Clin. Pharmacol. Ther. 35 (9), 374–380. 9314090

[B27] Welling P. G. Tse F. L. (1982). The influence of food on the absorption of antimicrobial agents. J. Antimicrob. Chemother. 9 (1), 7–27. 10.1093/jac/9.1.7 7037731

[B28] Williams L. Hill D. P. Davis J. A. Lowenthal D. T. (1996). The influence of food on the absorption and metabolism of drugs: An update. Eur. J. Drug Metab. Pharmacokinet. 21 (3), 201–211. 10.1007/BF03189714 8980916

[B29] Williams P. E. Harding S. M. (1984). The absolute bioavailability of oral cefuroxime axetil in male and female volunteers after fasting and after food. J. Antimicrob. Chemother. 13 (2), 191–196. 10.1093/jac/13.2.191 6706890

[B30] Wilson R. (1993). Int. J. Antimicrob. Agents 2 (3), 185–198. 10.1016/0924-8579(93)90053-8 18611534

