# Supplementary material for: Pharmacokinetics and safety of the two oral cefaclor formulations in healthy chinese subjects in the fasting and postprandial states
Source: Front Pharmacol. 2022 Oct 5;13:1012294. doi: 10.3389/fphar.2022.1012294 (PMC9581244; doi:10.3389/fphar.2022.1012294)
Supplement: Supplementary file 1 [file DataSheet1.docx]

**Supplementary Information**

**Pharmacokinetics and safety of the two oral cefaclor formulations in healthy Chinese subjects in the fasting and postprandial states**

Xinyao Qu^1,†^, Qiaohuan Deng^1,†^, Ying Li^2^, Peng Li^3^, Guangwen Liu^1^, Yanli Wang^1^, Zhengzhi Liu^1^, Shuang Yu^1^, Yang Cheng^1^, Yannan Zhou^1^, Jiahui Chen^1^, Qing Ren^1^, Zishu Yu^1^, Zhengjie Su^1^, Yicheng Zhao^4^, Haimiao Yang^1,*^

†These authors contributed equally.

*Correspondence: Haimiao Yang, MD, Email: haimiaoyang@outlook.com.

**Supplementary materials.1**

**Inclusion criteria**

Participants who meet all of the following criteria can be enrolled in this study:

1. Subjects have full awareness of the content and process of the trial, as well as penitential adverse events of the drug, and voluntarily provided written informed consent;

2. Subjects can complete the trial as planned;

3. Subjects (including men) do not to become pregnant and agree to use reliable contraceptives from the date of signing the informed consent to 3 months after the end of the trial;

4. Male and female subjects aged 18-55;

5. Male subjects weighted ≥ 50 kg, female subjects weighted ≥ 45kg, and the body mass index (BMI) was18 kg/m^2^ to 28 kg/m^2^;

6. Subject has no medical history of heart, liver, kidney, digestive tract, nervous system, mental disorders and metabolic disorders.

**Exclusion criteria**

Those who have one of the following conditions will be exclude from the study:

1. Subjects have a history of neurological and mental, respiratory, cardiovascular, digestive, blood and lymph, liver and kidney, endocrine, musculoskeletal and other diseases or other medical history that researchers consider may have the potential affect the drug metabolism or safety;

2. Subjects smoked at least 5 cigarettes within 3 months before screening;

3. Subjects have a history of special allergies (asthma, urticaria, eczema, etc.) or allergies (allergic to two or more drugs, food, milk and pollen), or hypersensitivity to the components of these drugs, cephalosporins and penicillin;

4. Subjects have alcohol abuse within a year (14 units of alcohol per week: one unit = 285 ml of beer, 24 of spirits, 100ml of wine);

5. Subjects donated or lost blood (> 400 ml) within 2 months before taking study drugs;

6. Subjects with a history of swallow hardly or other gastrointestinal disease that may affect drug metabolism;

7. Subjects have taken other prescription drugs within 14 days before taking study drugs;

8. Subjects have taken other non-prescription drugs, herbal or health products within 24 hours before taking study drugs;

9. Subjects ate special food (including grapefruit), or significantly changed their exercise habits, or other factors may affect drugs absorption, distribution, metabolism and excretion, within 48 hours before taking study drugs;

10. Subjects have taken part in other clinical trials within 3 months before the trial;

11. Subjects have obvious abnormalities in physical examination, vital sign examination, electrocardiogram, chest X-ray or clinical laboratory examination;

12. Subjects tested positive for hepatitis B surface antigen, hepatitis C antibody, HIV antibody or syphilis;

13. Subjects ate chocolate, any food and drink containing caffeine or xanthine within 48 hours before taking the drug;

14 Subjects took anything containing alcohol within 48 hours before taking the drug, or were tested positive alcohol;

15. The results of the test for and drugs perform positive or have a history of drug abuse in the past 5 years;

16. Female subjects are being in lactation or result of pregnancy test perform positive;

17. Subjects afraid of needles or the sight of blood;

18. Difficulty taking blood or intolerance to vein detained needle;

19. Subjects cannot respect the ward management regulations;

20. Subjects had serious disease or received major surgeries within 4 weeks before the trial;

21. Subjects have a history of postural hypotension;

22. Female subjects’ oral contraceptive pill within 30 days before the trial;

23. Female subjects used long-acting estrogen, progestin injections or implants within 6 months before the trial;

24. Subjects cannot complete the trial with personal reasons;

25. Researchers believe that it is not suitable in this trial for other reasons.

**Supplementary table. 1.** Major pharmacokinetic parameters C_max_, AUC_0-t_ and AUC_0-∞_ were transformed by natural logarithm and tested for significance by ANOVA.

|  | | *p* value | | |
| --- | --- | --- | --- | --- |
|  |  | Administration sequence | Administration period | Formulations |
| LnC_max_ | Fasting  (N = 22) | 0.0889 | 0.8345 | 0.1282 |
|  | Postprandial  (N = 24) | 0.1784 | 0.4965 | 0.0324 |
| LnAUC_0-t_ | Fasting  (N = 22) | 0.0077 | 0.7345 | 0.0237 |
|  | Postprandial  (N = 24) | 0.3307 | 0.2088 | 0.0025 |
| LnAUC_0-∞_ | Fasting  (N = 22) | 0.0074 | 0.7653 | 0.0252 |
|  | Postprandial  (N = 24) | 0.3467 | 0.2689 | 0.0034 |

N: number of subjects.

**Supplementary table. 2.** Blood concentration of Cefaclor granulefor subjects under the postprandial state (ng/mL).

| Subjetct | PER |  | C1 | C2 | C3 | C4 | C5 | C6 | C7 | C8 | C9 | C10 | C11 | C12 | C13 | C14 | C15 | C16 | C17 | C18 |
| --- | --- | --- | --- | --- | --- | --- | --- | --- | --- | --- | --- | --- | --- | --- | --- | --- | --- | --- | --- | --- |
| C001 | 1 | T | 0 | 0 | 16.9 | 38.6 | 49.0 | 60.6 | 77.1 | 101 | 207 | 521 | 1990 | 2480 | 1640 | 1580 | 1220 | 150 | 25.3 | 0 |
| C002 | 1 | T | 0 | 126 | 547 | 1100 | 943 | 708 | 571 | 547 | 723 | 935 | 1080 | 1140 | 937 | 699 | 896 | 265 | 28.5 | 0 |
| C003 | 2 | T | 0 | 0 | 0 | 20.2 | 45.6 | 57.8 | 104 | 1790 | 1110 | 853 | 1040 | 1940 | 1090 | 1100 | 1100 | 114 | 18.3 | 0 |
| C004 | 2 | T | 0 | 0 | 135 | 492 | 549 | 705 | 614 | 972 | 1170 | 1710 | 1740 | 1600 | 1430 | 979 | 723 | 105 | 28.4 | 0 |
| C005 | 1 | T | 0 | 10.4 | 78.1 | 167 | 210 | 218 | 261 | 597 | 1150 | 1440 | 1640 | 2050 | 2150 | 1490 | 818 | 74.6 | 13.5 | 0 |
| C006 | 1 | T | 0 | 442 | 1180 | 1620 | 1690 | 1380 | 1220 | 1160 | 972 | 1110 | 1040 | 1050 | 1020 | 834 | 741 | 122 | 56.6 | 61.0 |
| C007 | 2 | T | 0 | 189 | 1070 | 1760 | 1340 | 1100 | 925 | 1060 | 1190 | 1540 | 1200 | 858 | 720 | 542 | 250 | 26.7 | 0 | 0 |
| C008 | 2 | T | 0 | 751 | 3580 | 4190 | 3220 | 2430 | 1990 | 1500 | 1170 | 846 | 680 | 637 | 523 | 525 | 371 | 60.9 | 17.9 | 0 |
| C009 | 1 | T | 0 | 39.8 | 293 | 523 | 446 | 336 | 282 | 228 | 231 | 275 | 515 | 1430 | 1640 | 1600 | 846 | 270 | 98.3 | 43.5 |
| C010 | 1 | T | 0 | 322 | 1620 | 2690 | 2470 | 1850 | 1500 | 1350 | 1290 | 1120 | 852 | 772 | 540 | 567 | 478 | 144 | 39.9 | 0 |
| C011 | 2 | T | 0 | 0 | 12.3 | 36.1 | 55.6 | 93.6 | 166 | 362 | 530 | 984 | 1120 | 2160 | 2110 | 1720 | 1260 | 94.4 | 15.2 | 0 |
| C012 | 2 | T | 0 | 91.0 | 661 | 1190 | 1200 | 1570 | 1800 | 1850 | 1610 | 1180 | 1040 | 884 | 840 | 588 | 784 | 320 | 140 | 69.9 |
| C013 | 1 | T | 0 | 31.2 | 339 | 459 | 441 | 442 | 407 | 738 | 1150 | 1330 | 1120 | 891 | 640 | 974 | 836 | 187 | 41.4 | 11.3 |
| C014 | 2 | T | 0 | 132 | 1510 | 1730 | 1280 | 1300 | 1150 | 1020 | 1210 | 1220 | 1190 | 1160 | 1160 | 950 | 893 | 345 | 129 | 70.9 |
| C015 | 1 | T | 0 | 278 | 878 | 926 | 839 | 688 | 492 | 1240 | 1280 | 1540 | 1310 | 1170 | 1260 | 900 | 464 | 54.6 | 12.3 | 0 |
| C016 | 2 | T | 0 | 49.2 | 639 | 1450 | 1540 | 1360 | 1150 | 1020 | 1000 | 942 | 1500 | 1910 | 1430 | 1080 | 536 | 73.0 | 12.2 | 0 |
| C017 | 1 | T | 0 | 0 | 10.4 | 35.6 | 38.5 | 59.4 | 109 | 262 | 356 | 472 | 984 | 1010 | 1310 | 1960 | 1370 | 343 | 85.6 | 13.9 |
| C018 | 2 | T | 0 | 239 | 1030 | 1410 | 1200 | 932 | 874 | 1080 | 1300 | 1210 | 1430 | 1400 | 1110 | 999 | 581 | 54.8 | 10.9 | 0 |
| C019 | 2 | T | 0 | 42.6 | 616 | 1850 | 1620 | 1150 | 944 | 714 | 520 | 425 | 381 | 371 | 528 | 800 | 797 | 245 | 60.4 | 28.3 |
| C020 | 1 | T | 0 | 44.5 | 495 | 1440 | 1880 | 1870 | 1760 | 2040 | 1880 | 1700 | 1400 | 1230 | 1030 | 768 | 689 | 131 | 32.9 | 0 |
| C021 | 2 | T | 0 | 115 | 1460 | 2270 | 1810 | 1360 | 1270 | 989 | 966 | 1130 | 1380 | 1100 | 1180 | 796 | 454 | 50.2 | 10.0 | 0 |
| C022 | 1 | T | 0 | 0 | 19.3 | 37.1 | 60.1 | 112 | 130 | 170 | 265 | 398 | 610 | 892 | 977* | 1510 | 1600 | 218 | 67.6 | 26.9 |
| C023 | 1 | T | 0 | 26.8 | 279 | 741 | 1300 | 1920 | 1700 | 1690 | 1390 | 1290 | 1070 | 984 | 1060 | 727 | 800 | 185 | 47.1 | 12.8 |
| C024 | 2 | T | 0 | 1200 | 2570 | 2410 | 1810 | 1470 | 1230 | 1470 | 1450 | 1410 | 1330 | 1300 | 1280 | 974 | 712 | 137 | 32.8 | 0 |

PER: administration period; T: test drug; C1: blood was collected within 60 min (pre-dose); C2: blood was collected at 5 min; C3: blood was collected at 10 min; C4: blood was collected at 20 min; C5: blood was collected at 30 min; C6: blood was collected at 45 min; C7: blood was collected at 1 h; C8: blood was collected at 1 h 20 min; C9: blood was collected at 1 h 40 min; C10: blood was collected at 2 h; C11: blood was collected at 2 h 20 min; C12: blood was collected at 2 h 40 min; C13: blood was collected at 3 h; C14: blood was collected at 3.5 h; C15: blood was collected at 4 h; C16: blood was collected at 6 h; C17: blood was collected at 8 h; C18: blood was collected at 10 h.

**Supplementary table. 3.** Blood concentration of Cefaclor suspension for subjects under the postprandial state (ng/mL).

| Subject | PER |  | C1 | C2 | C3 | C4 | C5 | C6 | C7 | C8 | C9 | C10 | C11 | C12 | C13 | C14 | C15 | C16 | C17 | C18 |
| --- | --- | --- | --- | --- | --- | --- | --- | --- | --- | --- | --- | --- | --- | --- | --- | --- | --- | --- | --- | --- |
| C001 | 2 | R | 0 | 0 | 0 | 0 | 0 | 0 | 0 | 54.9 | 269 | 1130 | 3980 | 2970 | 2030 | 1340 | 712 | 84.3 | 16.2 | 0 |
| C002 | 2 | R | 0 | 23.2 | 111 | 729 | 631 | 593 | 524 | 762 | 889 | 1070 | 1260 | 917 | 878 | 1080 | 922 | 154 | 63.9 | 0 |
| C003 | 1 | R | 0 | 0 | 0 | 27.4 | 91.8 | 148 | 188 | 459 | 804 | 2080 | 1610 | 1530 | 1220 | 1080 | 879 | 132 | 23.0 | 0 |
| C004 | 1 | R | 0 | 120 | 608 | 938 | 896 | 720 | 660 | 513 | 541 | 801 | 1490 | 1710 | 1520 | 1640 | 995 | 113 | 29.6 | 0 |
| C005 | 2 | R | 0 | 45.2 | 171 | 272 | 284 | 267 | 338 | 618 | 824 | 1050 | 1530 | 2450 | 2770 | 1470 | 748 | 74.8 | 14.1 | 0 |
| C006 | 2 | R | 0 | 55.5 | 775 | 1210 | 1770 | 1760 | 1620 | 1580 | 1230 | 1020 | 981 | 970 | 826 | 769 | 633 | 88.2 | 56.3 | 37.7 |
| C007 | 1 | R | 0 | 79.3 | 345 | 708 | 806 | 719 | 658 | 815 | 1170 | 1780 | 1600 | 1350 | 1040 | 655 | 326 | 36.2 | 0 | 0 |
| C008 | 1 | R | 0 | 1010 | 2720 | 3980 | 2910 | 2300 | 2060 | 1750 | 1380 | 1040 | 894 | 761 | 629 | 485 | 338 | 68.4 | 24.4 | 0 |
| C009 | 2 | R | 0 | 31.0 | 296 | 592 | 481 | 332 | 272 | 330 | 556 | 486 | 744 | 1800 | 1770 | 1050 | 935 | 240 | 96.5 | 65.1 |
| C010 | 2 | R | 0 | 493 | 2280 | 2950 | 2270 | 1600 | 1300 | 1130 | 977 | 926 | 851 | 821 | 894 | 749 | 516 | 58.8 | 25.3 | 0 |
| C011 | 1 | R | 0 | 0 | 12.2 | 46.2 | 84.2 | 127 | 155 | 225 | 375 | 551 | 1070 | 1540 | 1860 | 2430 | 1760 | 149 | 28.3 | 0 |
| C012 | 1 | R | 0 | 984 | 2600 | 2410 | 1910 | 1820 | 1160 | 1350 | 1120 | 1070 | 996 | 787 | 790 | 871 | 643 | 313 | 110 | 20.8 |
| C013 | 2 | R | 0 | 24.1 | 88.1 | 134 | 150 | 226 | 360 | 730 | 964 | 1060 | 1240 | 1200 | 1190 | 1220 | 987 | 107 | 31.7 | 11.7 |
| C014 | 1 | R | 0 | 91.3 | 1680 | 2840 | 2570 | 2000 | 1460 | 1380 | 1060 | 975 | 882 | 874 | 1070 | 664 | 853 | 286 | 181 | 74.2 |
| C015 | 2 | R | 0 | 717 | 1780 | 1850 | 1330 | 1040 | 885 | 776 | 1050 | 1210 | 1490 | 1270 | 1080 | 845 | 452 | 49.2 | 10.2 | 0 |
| C016 | 1 | R | 0 | 28.4 | 410 | 1540 | 1700 | 1760 | 1490 | 1470 | 1140 | 958 | 1050 | 1530 | 1830 | 1210 | 681 | 74.8 | 20.1 | 0 |
| C017 | 2 | R | 0 | 0 | 0 | 0 | 0 | 0 | 0 | 88.5 | 473 | 1040 | 1130 | 1460 | 1770 | 1540 | 1420 | 255 | 66.5 | 14.2 |
| C018 | 1 | R | 0 | 93.6 | 1310 | 1790 | 1510 | 1100 | 940 | 1340 | 1430 | 1230 | 1230 | 1270 | 1040 | 833 | 675 | 79.0 | 24.0 | 0 |
| C019 | 1 | R | 0 | 92.4 | 1340 | 3010 | 2600 | 1770 | 1580 | 1140 | 964 | 703 | 676 | 589 | 561 | 617 | 412 | 79.1 | 22.3 | 0 |
| C020 | 2 | R | 0 | 254 | 1550 | 2760 | 2810 | 2300 | 1710 | 1630 | 1310 | 1140 | 1020 | 1050 | 927 | 715 | 607 | 116 | 34.3 | 10.7 |
| C021 | 1 | R | 0 | 678 | 3790 | 2880 | 2200 | 1660 | 1230 | 1250 | 1240 | 1270 | 1130 | 1080 | 748 | 625 | 395 | 34.6 | 0 | 0 |
| C022 | 2 | R | 0 | 0 | 52.6 | 199 | 231 | 278 | 328 | 452 | 540 | 820 | 1310 | 1190 | 1170 | 1250 | 1270 | 144 | 45.3 | 12.9 |
| C023 | 2 | R | 0 | 17.1 | 230 | 816 | 834 | 785 | 881 | 1040 | 1010 | 1020 | 1410 | 1340 | 1330 | 1510 | 1070 | 243 | 65.0 | 15.5 |
| C024 | 1 | R | 0 | 216 | 918 | 1070 | 1130 | 923 | 1080 | 1450 | 1440 | 1590 | 1750 | 1770 | 1380 | 1250 | 799 | 184 | 52.7 | 14.7 |

PER: administration period; R: reference drug; C1: blood was collected within 60 min (pre-dose); C2: blood was collected at 5 min; C3: blood was collected at 10 min; C4: blood was collected at 20 min; C5: blood was collected at 30 min; C6: blood was collected at 45 min; C7: blood was collected at 1 h; C8: blood was collected at 1 h 20 min; C9: blood was collected at 1 h 40 min; C10: blood was collected at 2 h; C11: blood was collected at 2 h 20 min; C12: blood was collected at 2 h 40 min; C13: blood was collected at 3 h; C14: blood was collected at 3.5 h; C15: blood was collected at 4 h; C16: blood was collected at 6 h; C17: blood was collected at 8 h; C18: blood was collected at 10 h.

**Supplementary table. 4.** The plasma concentrations of samples exceeded the linear quantitative range from 10.0 ng/mL to 8000 ng/ mL.

| Cefaclor granule | | Cefaclor suspension | |
| --- | --- | --- | --- |
| Subject | Concentration (ng/mL) | Subject | Concentration (ng/mL) |
| K005 | 8390 | K013 | 11500 |
| K020 | 9430 | K017 | 10100 |
| K022 | 9380 | K020 | 10800 |
| - | - | K022 | 9030 |

All exceeded linear quantitative range samples happened at the C4 sample point (20 min after administration).

**Supplementary Figure.1** PK analysis of Cefaclor granule and Cefaclor suspension in in the postprandial state for each subject.


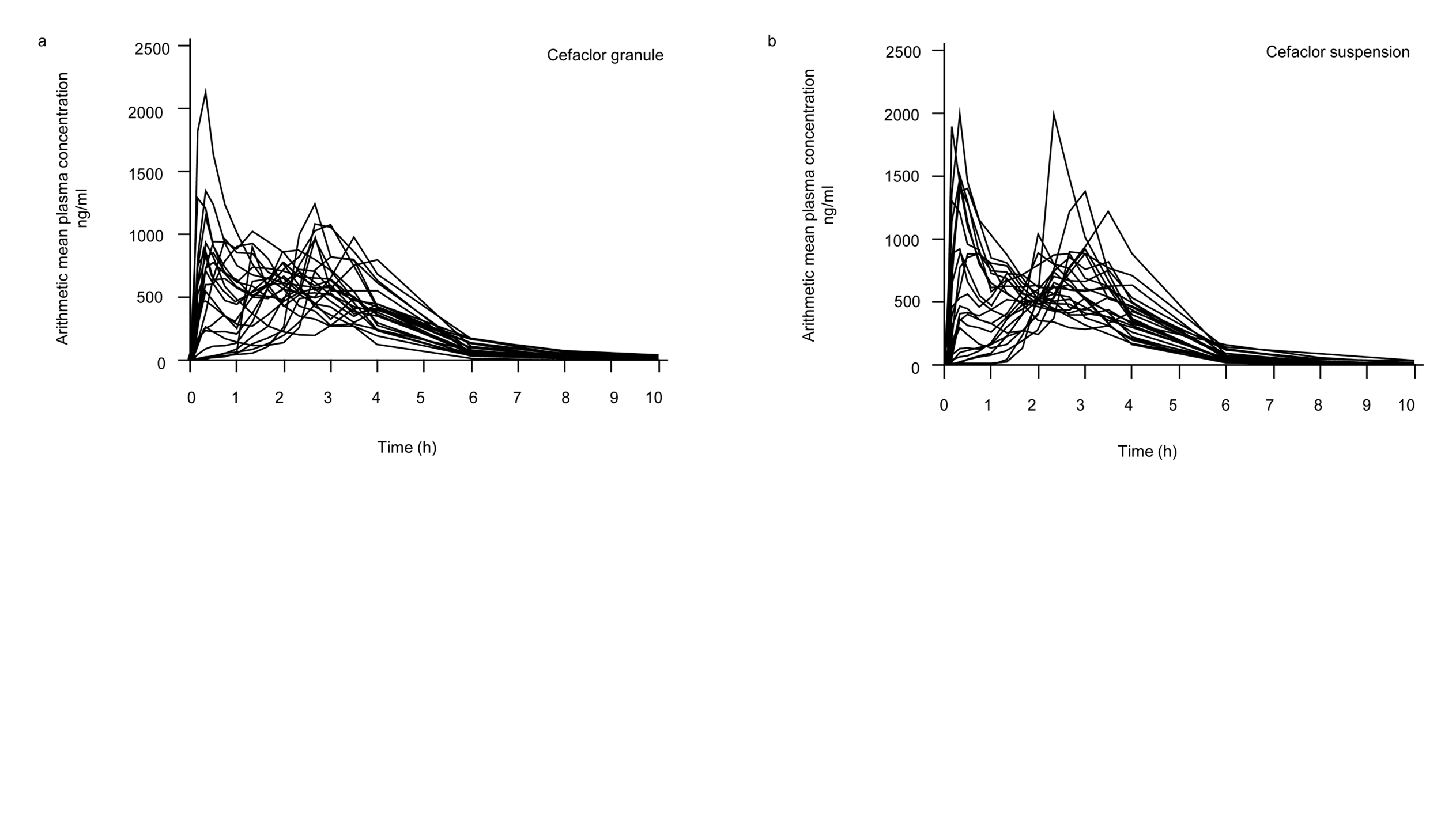


Mean blood concentration time curve after oral administrated cefaclor for each subject (a); Mean blood concentration time curve after oral administrated Cefaclor suspension for each subject (b).
